# Supplementary figures and images for: Secreted filarial nematode galectins modulate host immune cells
Source: Front Immunol. 2022 Aug 11;13:952104. doi: 10.3389/fimmu.2022.952104 (PMC9402972; doi:10.3389/fimmu.2022.952104)

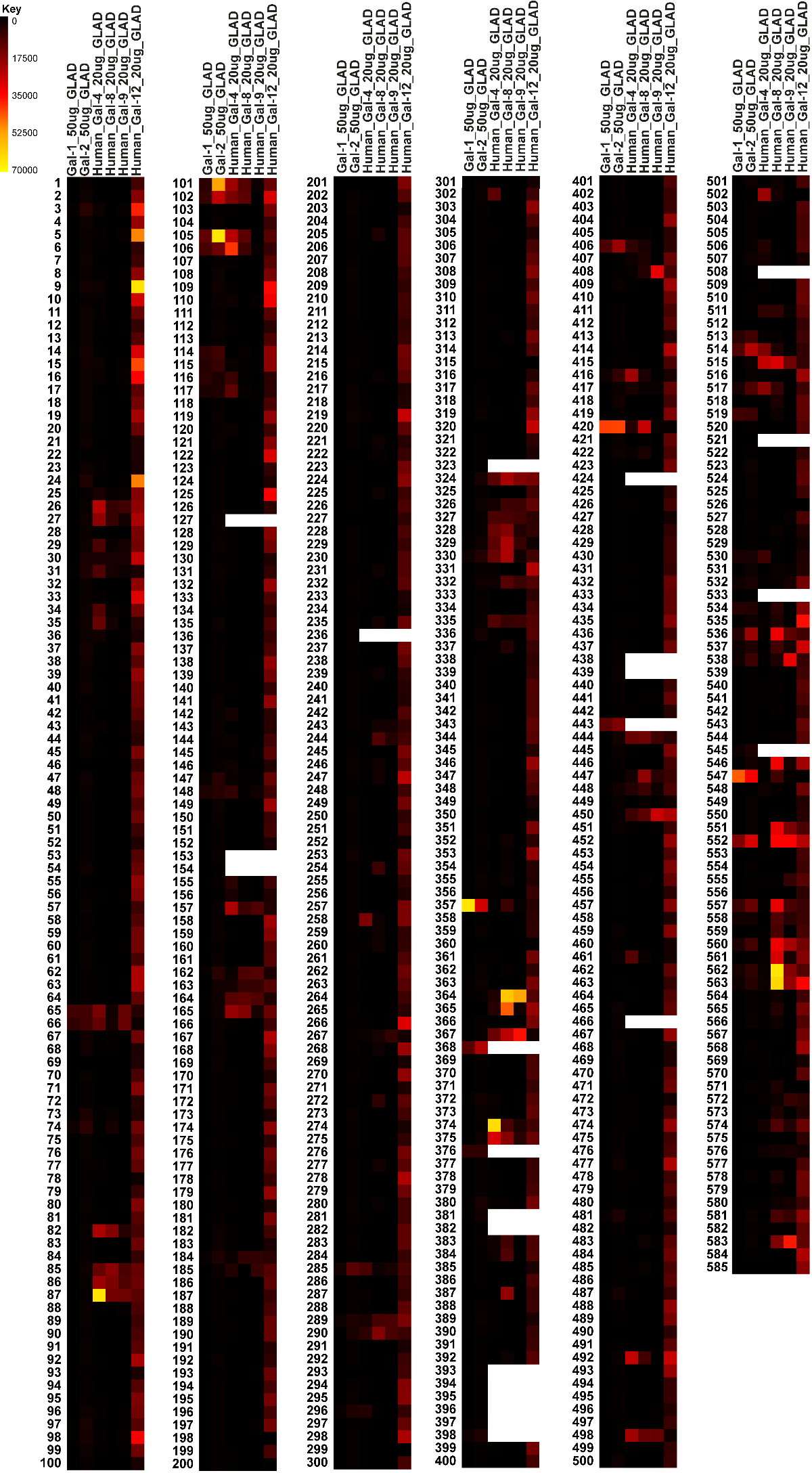

Supplement: Supplementary file 2 [file DataSheet_3.docx]
