# Supplementary material for: Secreted filarial nematode galectins modulate host immune cells
Source: Front Immunol. 2022 Aug 11;13:952104. doi: 10.3389/fimmu.2022.952104 (PMC9402972; doi:10.3389/fimmu.2022.952104)
Supplement: Supplementary file 4 [file DataSheet_5.docx]

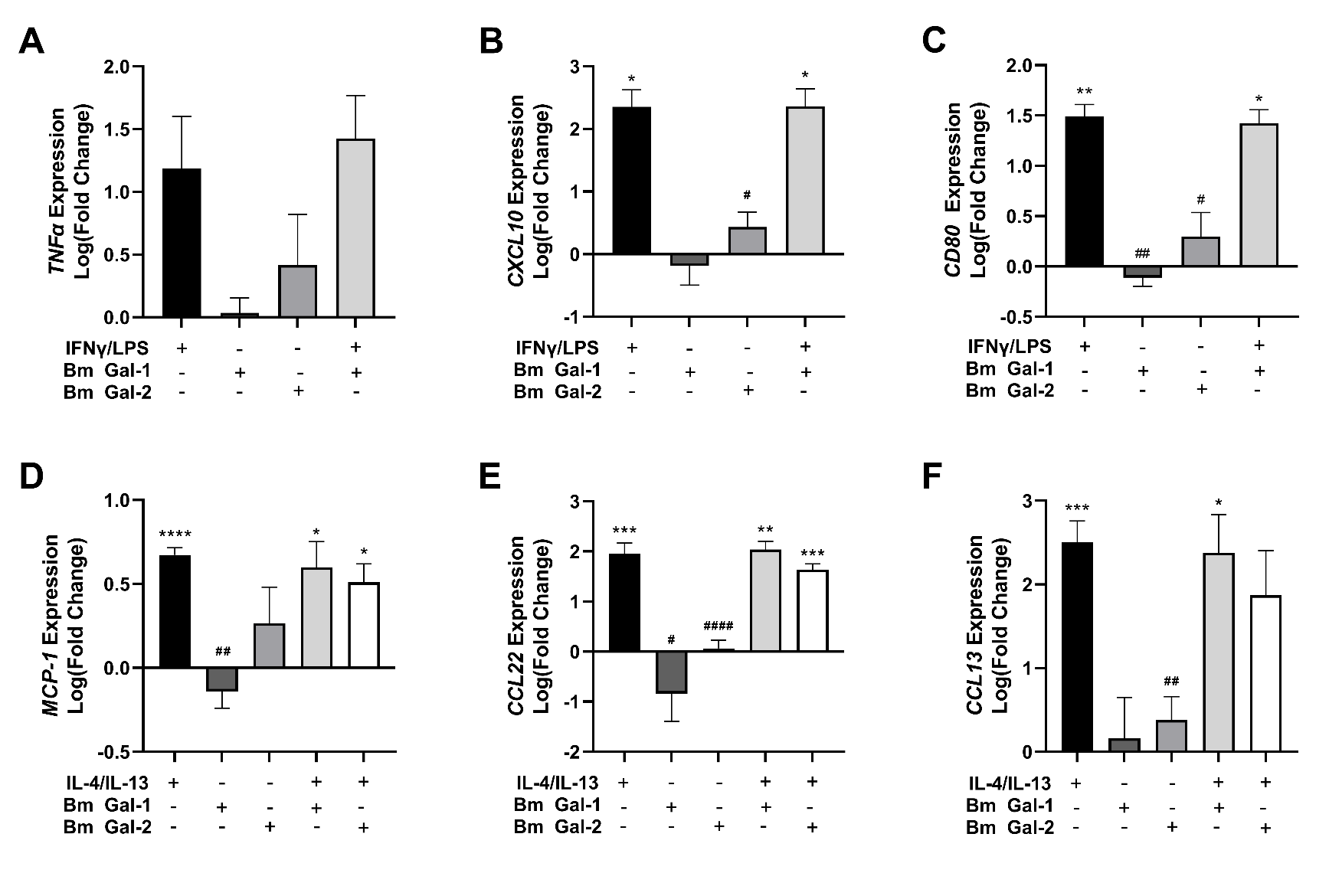


**Supplemental Materials 5. rBma-LEC-1 and rBma-LEC-2 do not increase M1 markers and some M2 markers**

rBma-LEC-1 and rBma-LEC-2 did not statistically increase any of the M1 markers tested (A-C). While rBma-LEC-2 did statistically increase IL-10, it did not statistically increase other M2 markers that were tested including *MCP-1, CCL22, and CCL13* (D-F). N = 3 (minimum). Mean ± SEM, * = Statistical significance relative to M0. *P<0.05, **P<0.01, ***P<0.001, ****P<0.0001. # = Statistical significance relative to M2. #P<0.05, ##P<0.01, ####P<0.0001.
