# Supplementary material for: Secreted filarial nematode galectins modulate host immune cells
Source: Front Immunol. 2022 Aug 11;13:952104. doi: 10.3389/fimmu.2022.952104 (PMC9402972; doi:10.3389/fimmu.2022.952104)
Supplement: Supplementary file 5 [file DataSheet_6.docx]

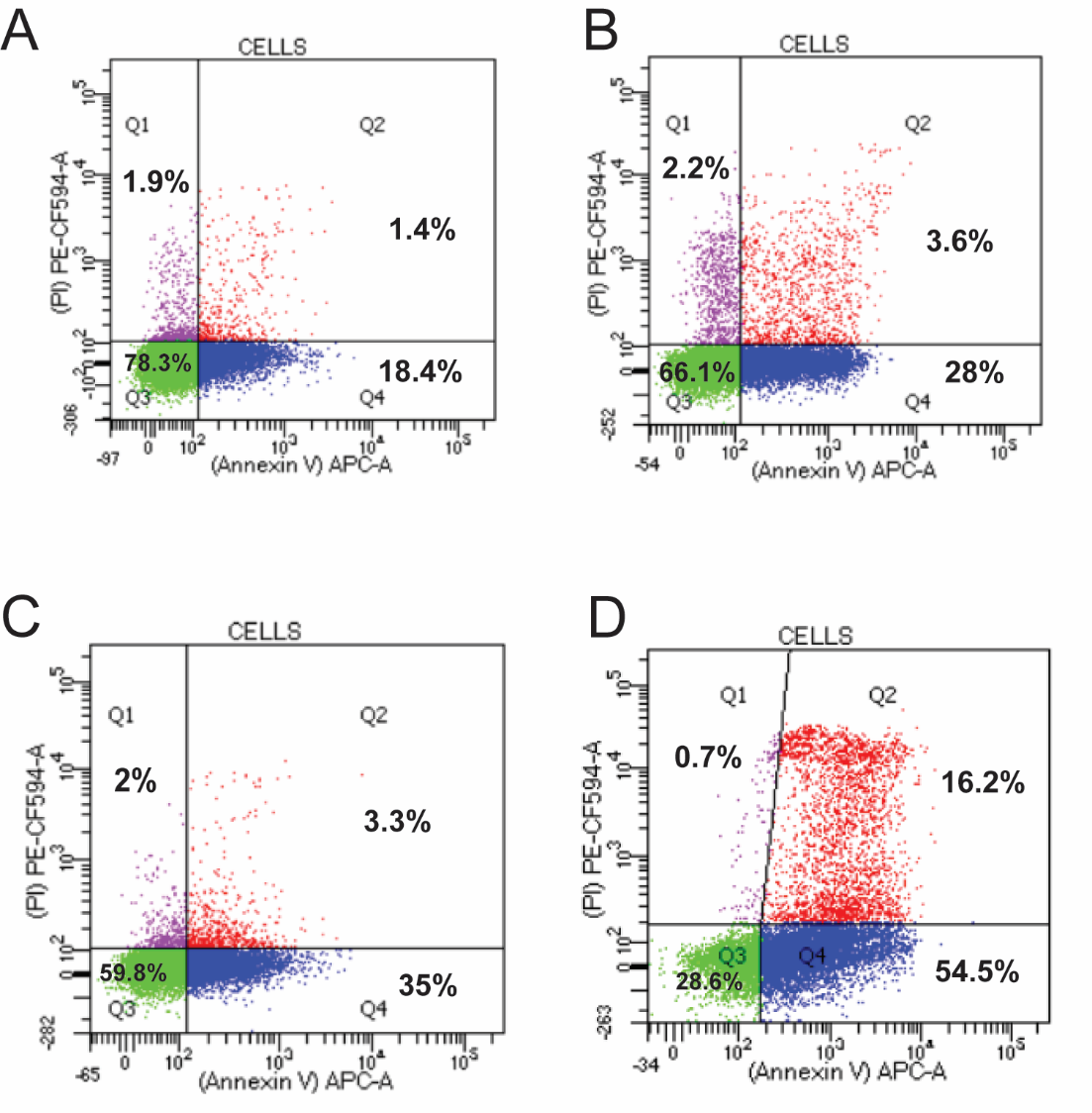


**Supplemental Materials 6. Annexin V Flow Cytometry Plots**

Naïve CD4^+^ T cells were isolated from C57BL/6 WT mice. Naïve and T cells polarized to Th1 were treated with either dPBS or rBma-LEC-2. Cells were stained using the eBioscience Annexin V Apoptosis Detection Kit APC and analyzed for apoptosis by flow cytometry. Flow cytometry plots for (A) Th0 negative control, (B) Th0 rBma-LEC-2, (C) Th1 negative control and (D) Th1 rBma-LEC-2. Quadrant 2 and quadrant 4 are considered apoptotic cells. Quadrant 3 are considered healthy cells. Quadrant 1 are considered cells that were dead prior to treatment and were excluded from the analysis.
