# Supplementary material for: Secreted filarial nematode galectins modulate host immune cells
Source: Front Immunol. 2022 Aug 11;13:952104. doi: 10.3389/fimmu.2022.952104 (PMC9402972; doi:10.3389/fimmu.2022.952104)
Supplement: Supplementary file 6 [file Table_1.pdf]

Table 1. Primer Sequences

Experiment	Gene/Protein	Forward Primer	Reverse Primer
Protein Expression			
	Bma-lec-1 (WBGene00226528)	5’AAAGGATCCATGTCTGATCAGAG ATCATATCC3’	5’AAAGCGGCCGCGTGAATTTGTATGCC CGTAAG3’
	Bma-lec-2 (WBGene00224538)	5’AAAAAGCTTATGGCCAATGAATATG AAACGAATTATCC-3’	5’AAAGCGGCCGCGCGCATCTGGATACC GCTTAC3’
Galectin Expression across Life stages qPCR			
	Bma-lec-1	5’-CTGCCATTGTCTTCCGTATCT-3’	5’-TGCCTTCCCTCTCCTCATTA-3’
	Bma-lec-2	5’- ACGCTTCCACATCAATCTACTC-3’	5’-GCATCTGGATACCGCTTACTT-3’
	B. malayi NADH Dehydrogenase Subunit 1 (NC_004298.1)	5’-GGGTGGCACTCAGTGTCGTA-3’	5’-ACAACGCCTGAAAAATACCAG-3’
Macrophage Polarization			
	CCL13	5’-CCAAACTGGGCAAGGAGAT-3’	5’GTCTTCAGGGTGTGAGCTTT-3’
	CCL22	5’-TAGGCTCTTCATTGGCTCAG-3’	5’-ATTACGTCCGTTACCGTCTG-3’

	CD80	5’-ATCCTGGGCCATTACCTTAATC-3’	5’-CTCTCATTCCTCCTTCTCTCTCT-3’
	CXCL10	5’-CCATTCTGATTTGCTGCCTT-3’	5’-TACTAATGCTGATGCAGGTA-3’
	IL10	5’-TACGGCGCTGTCATCGATTT-3’	5’-TAGAGTCGCCACCCTGATGT-3’
	MCP1(CCL2)	5’-GATCTCAGTGCAGAGGCTCG-3’	5’-TTTGCTTGTCCAGGTGGTCC-3’
	RPL37A	5’-ATTGAAATCAGCCAGCACGC-3’	5’-AGGAACCACAGTGCCAGATCC-3’
	TNF	5’-TCTTCTCGAACCCCGAGTGAC-3’	5’-TTTGCTTGTCCAGGTGGTCC-3’
